# Case Report: Progressive disease of BRCA2-mutant colon adenocarcinoma following talazoparib therapy

**DOI:** 10.3389/fonc.2023.1245547

**Published:** 2023-11-07

**Authors:** Elizaveta Polyanskaya, Alexandra Lebedeva, Olesya Kuznetsova, Ekaterina Belova, Alexandra Kavun, Maxim Ivanov, Mikhail Fedyanin, Alexey Tryakin, Vladislav Mileyko, Dmitry Nosov

**Affiliations:** ^1^ Department of Clinical Pharmacology and Chemotherapy #2, N.N. Blokhin Russian Cancer Research Center, Moscow, Russia; ^2^ RnD, OncoAtlas LLC, Moscow, Russia; ^3^ Faculty of Physics, Lomonosov Moscow State University, Moscow, Russia; ^4^ Phystech School of Biological and Medical Physics, Moscow Institute of Physics and Technology, Dolgoprudny, Russia; ^5^ State Budgetary Institution of Healthcare of the City of Moscow “Moscow Multidisciplinary Clinical Center “Kommunarka”, Department of Health of the City of Moscow, Moscow, Russia; ^6^ Federal State Budgetary Institution “National Medical and Surgical Center Named After N.I. Pirogov”, Ministry of Health of the Russian Federation, Moscow, Russia; ^7^ Oncology Department of Antitumor Pharmacological Therapy (with Day Hospital), The Central Clinical Hospital of the Administrative Directorate of the President of the Russian Federation, Moscow, Russia

**Keywords:** colorectal cancer, BRCA2, talazoparib, PARP inhibitors, MTB, case report

## Abstract

Colorectal cancer (CRC) is currently one of the most common tumor types diagnosed worldwide. In the early stages, the disease responds well to surgical and chemotherapeutic treatment, but in the later stages when therapeutic options are exhausted, comprehensive genomic profiling can guide further treatment decisions. We present the case of a 46-year-old man of Ashkenazi Jewish ancestry who was diagnosed with KRAS-mutated metastatic colorectal cancer. After surgery and progression on standard FOLFOX/FOLFIRI + bevacizumab therapy, as well as on Trifluridine/Tipiracil, comprehensive genomic profiling was performed with the hope of expanding therapeutic options. Following comprehensive tumor molecular profiling via NGS, a discussion of the case was discussed at the local molecular tumor board in order to determine further treatment strategy. An activating variant of KRAS and PIK3CA, FLT3 and SRC amplification and damaging TP53 and APC variants were discarded by MTB as potential targetable biomarkers. The BRCA2 p.S1415fs*4 founder frameshift variant was of interest and the patient was included in the clinical trial investigating the efficacy of a PARP inhibitor talazoparib. Unfortunately, the disease progression was detected within one month of talazoparib treatment and the patient died during the 8th cycle of FOLFIRI + bevacizumab therapy rechallenge.

## 1 Introduction

Colorectal cancer (CRC) is currently the third most common diagnosed malignancy worldwide. More than 1.9 million new cases of colorectal cancer and more than 0.9 million deaths due to colorectal cancer were estimated in 2020. In the early stage, the prognosis for patients is favorable, but only a small percentage of patients with stage IV disease achieve a 5-year survival. Chemotherapy or targeted drugs guided by the molecular alterations are used as the initial therapy for patients with metastatic colorectal cancer. However, in cases when suitable therapeutic options are exhausted, comprehensive genomic profiling can guide further treatment decisions. Up to 15% of CRC patients have mutations in HRR genes (1). Deleterious mutations in the BRCA1/2 genes are a well-established biomarker for PARP inhibitor treatment, and this approach is being actively explored in various types of cancer (2). To date, no randomized clinical trials have assessed the efficacy of PARP inhibitors for the treatment of BRCA-mutated CRC. Here we present the case of a heavily pretreated 46-year-old male patient who was diagnosed with CRC and was found to harbor a germline BRCA2 founder mutation, who was later treated with PARP inhibitor talazoparib.

## 2 Case description

А 46-year-old man of Ashkenazi Jewish ancestry was diagnosed with metastatic colorectal cancer (sigmoid) in 2020 and was firstly treated in the local hospital. He did not have any chronic diseases or family history of colorectal or other malignancies. In 2017, the patient was treated surgically for adenoma of salivary gland. The patient used to be a smoker, however, he stopped in 2011. Following primary molecular testing, KRAS p.G12A mutation was detected, and the tumor had MSS status. The tumor was moderately differentiated adenocarcinoma, R0. Due to metastatic spread to liver and lungs and the presence of a KRAS mutation, which was uncovered following conventional PCR testing, first line therapy with FOLFOX + bevacizumab was initiated. Of note, the tumor had MSS status. After 6 cycles, the patient presented with abdominal pain. CT scan revealed rectovesical fistula formation, block of the left ureter, partial intestinal obstruction, and the size of lung and liver lesions remained stable with tendency to partial response. The patient presented to N.N. Blokhin Cancer Research Center (Moscow, Russia) for further treatment. In June 2020, the patient underwent resection of sigmoid, rectum, posterior wall of the bladder, left ureter. He continued to receive FOLFOX + bevacizumab for another 6 cycles. After a total of 12 cycles of first-line therapy disease progression was observed. A lesion in the brain was detected. The patient underwent stereotactic therapy, and on another CT new lesions in lungs, left adrenal gland were detected. Second line treatment with FOLFIRI + bevacizumab was recommended. After receiving 15 cycles, disease progression was detected (increase of lung and liver lesions, stable brain lesions) and the patient was suggested to take part in a clinical trial of Trifluridine/Tipiracil. The patient received 6 months of Trifluridine/Tipiracil with maximum effect of stable disease. In December 2021 disease progression was observed (lung lesions).

In January 2021, comprehensive tumor molecular profiling via NGS was performed on a patient’s tumor sample using FoundationOne CDx. Genomic analysis revealed common oncogenic variants KRAS p.G12A and PIK3CA p.E545K, which have been reported to be frequently found in colon adenocarcinoma (3). Other typical findings for colon adenocarcinoma included two distinct variants in the APC gene (p.R1114* and p.S1415fs*4), FLT3 amplification and TP53 p.P151A. Additionally, SRC amplification and a BRCA2 p.S1982fs*22 variant were detected. Variant allele frequencies were not reported for either of the variants. Following disease progression, the patient was referred to a Molecular Tumor Board (MTB) at OncoAtlas to discuss potential treatment implications of the genomic findings.

**Figure 1:**
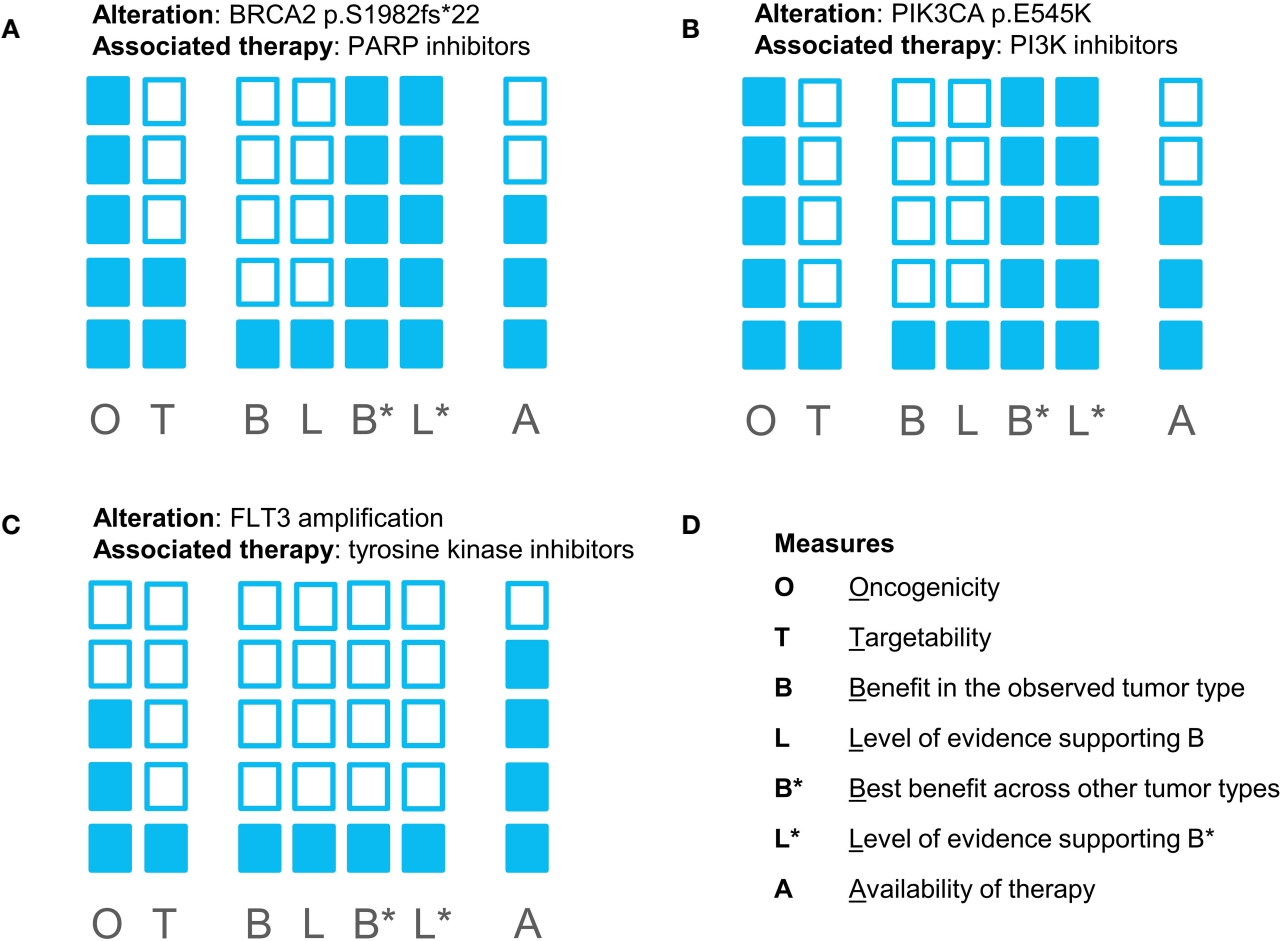
Evidence blocks supporting biomarker-drug associations for all potentially targetable alterations found in the patient’s tumor sample: **(A)** for a BRCA2 mutation and PARP inhibitors; **(B)** for a PIK3CA mutation and PI3K inhibitors; **(C)** for FLT3 amplification and tyrosine kinase inhibitors. **(D)** represents the measures reflected in the evidence blocks. The shading of more blocks reflects higher confidence in the evidence supporting the effect of alteration and the feasibility of targeting the biomarker.

Following discussion of the case at MTB (**Figure 1**), EGFR-targeting agents were ruled out as potential treatment options due to the presence of an oncogenic KRAS mutation. Despite the fact that the PIK3CA variant was indeed oncogenic, the MTB concluded that targeting this alteration with a PI3K inhibitor Alpelisib would have unlikely resulted in response to therapy due to the constitutively activated KRAS and the interplay between RAS and PI3K (4). Targeting the amplification of FLT3 by nonspecific tyrosine kinase inhibitors (e.g., sunitinib, sorafenib, regorafenib, etc.) failed to result in suitable clinical activity, as shown in multiple studies (5, 6). Thus, this alteration was not considered actionable by MTB. Finally, the BRCA2 p.S1415fs*4 frameshift variant was found, commonly referred to as c.6174delT, a pathogenic founder mutation that is prevalent in the Ashkenazi Jewish population (7), which is consistent with the patient’s self-reported ancestry. Although previously published evidence of using PARP inhibitors in colon cancer suggested that this approach might be suboptimal, the nature of this evidence was limited. At the time of MTB, a clinical trial investigating the efficacy of a PARP inhibitor talazoparib was recruiting patients with solid tumors harboring alterations in HRR genes. Thus, the patient started talazoparib therapy (standard dose, 1 mg/daily) as part of a clinical trial.

**Figure 2:**
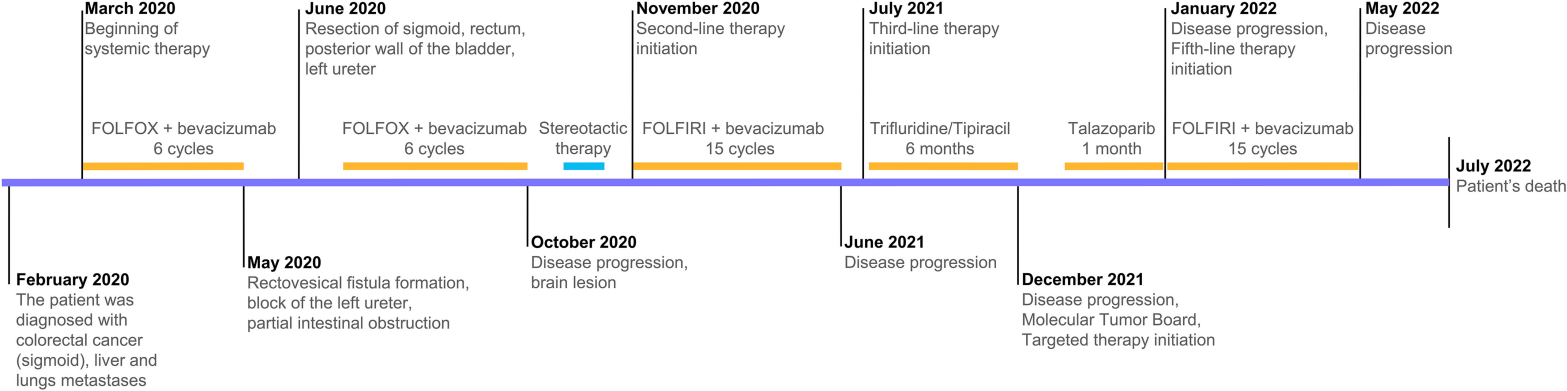
Treatment timeline.

Disease progression due to a ≥20% increase in the total diameter of the target lesions (liver, lungs, brain), as well as development of new lesions, was observed within one month of talazoparib treatment. Importantly, renal function was sustained both prior and following talazoparib treatment. The patient experienced no adverse events during talazoparib treatment. After that, rechallenge of FOLFIRI + bevacizumab was administered, the patient received 8 cycles of 5th line treatment and died within a month of the last cycle (**Figure 2**).

## 3 Discussion

Here, we describe a case of progressive disease following PARP inhibitor therapy in a patient with BRCA2-mutant colorectal adenocarcinoma. Since the patient has exhausted all of the standard therapeutic options, and a clinical trial was available, it was decided to administer experimental talazoparib therapy.

Germline mutations of BRCA1/2 are found in approximately 1% of patients affected with colorectal cancer (8). Several studies reported no significant risk increase of developing colorectal cancer among BRCA2 mutation carriers (9). To date, no large randomized clinical trials have evaluated the effect of PARP inhibitors for the treatment of BRCA-mutant colorectal cancer. However, the currently available evidence, consisting of mostly case reports and pan-cancer studies, might suggest that most cases of colorectal cancer are unlikely to respond to treatment with PARP inhibitors. Nonetheless, there have been no reports on the effects of talazoparib for colorectal cancer.

Several trials investigated the efficacy of PARP inhibitor therapy, in monotherapy or combined with chemotherapeutic agents in biomarker-unselected colorectal cancer patients. For instance, an ORR of 22.2% was observed in a study of investigational PARP inhibitor veliparib with concurrent FOLFIRI among patients with colorectal cancer (10). Similarly, a study of veliparib plus temozolomide demonstrated an DCR of 22.2%, including 2 (4%) partial responses (11). A case of stable disease observed in a colorectal cancer patient has been reported in a phase I trial of veliparib combined with topotecan in a pan-cancer study, while resulting in an ORR of 0% among patients with colorectal cancer (12). Consistently, two studies of olaparib in monotherapy or combined with topotecan for patients with colorectal reported an ORR of 0% (13, 14). Another phase II study of veliparib combined with FOLFIRI with or without bevacizumab compared with placebo reported no significant differences in ORR, PFS and OS between the two groups (15).

A few in vitro studies show that colorectal cancer cells harboring mutations in the HRR genes are sensitive to PARP inhibitors (16). However, results of basket clinical trials, as well as case reports, suggest that this approach might not translate to the clinical setting. For instance, among two patients included in the DRUP trial, none of the patients exhibited objective response or stable disease following olaparib treatment (17). Another basket study, TAPUR, demonstrated an ORR of 4% among patients with ATM-mutated colorectal cancer treated with olaparib, indicating the lack of potential efficacy of olaparib monotherapy (18). Results from the TAPUR cohort of patients with BRCA1/2-mutated solid tumors reported 2 disease stabilizations as best response for at least 16 weeks among two patients with colorectal cancer (19). A stable disease for almost 23 months was achieved for a colorectal cancer patient harboring a germline BRCA1 mutation treated with veliparib (20). Additionally, a case report of disease stabilization following off-label olaparib combined with irinotecan used for the treatment of ATM-deficient colorectal cancer patient with survival of 14 months (21). Altogether, the results of these studies indicate that PARP inhibitors have limited activity in the treatment of colorectal cancer, both in biomarker-unselected and HRR gene-mutated cases.

Despite encouraging antitumor activity demonstrated in a wide variety of tumor types (2), PARP inhibitors do not seem to hold any promise in altering the treatment landscape of colorectal cancer, both with and without features of HRD. Our case adds to the existing limited evidence on the lack of potential therapeutic benefit of PARP inhibitors for the treatment of colorectal cancer patients harboring germline pathogenic BRCA1/2 mutations. Furthermore, this is the first body of evidence reporting on the use of talazoparib for the treatment of colorectal cancer.

## Data availability statement

The original contributions presented in the study are included in the article/supplementary material. Further inquiries can be directed to the corresponding author.

## Ethics statement

Written informed consent was obtained from the individual(s) for the publication of any potentially identifiable images or data included in this article.

## Author contributions

EP, OK, MF and AT carried out the studies, participated in collecting data. AL, OK, MI performed data analysis. AL, OK, EB and AK drafted the manuscript. VM and DN provided administrative support. All authors contributed to the article and approved the submitted version.
